# Circulating Levels of Adiponectin, Leptin, Fetuin-A and Retinol-Binding Protein in Patients with Tuberculosis: Markers of Metabolism and Inflammation

**DOI:** 10.1371/journal.pone.0038703

**Published:** 2012-06-07

**Authors:** Naoto Keicho, Ikumi Matsushita, Takahiro Tanaka, Takuro Shimbo, Nguyen Thi Le Hang, Shinsaku Sakurada, Nobuyuki Kobayashi, Minako Hijikata, Pham Huu Thuong, Luu Thi Lien

**Affiliations:** 1 Department of Respiratory Diseases, Research Institute, National Center for Global Health and Medicine, Tokyo, Japan; 2 Department of Clinical Research and Informatics, International Clinical Research Center, National Center for Global Health and Medicine, Tokyo, Japan; 3 National Center for Global Health and Medicine–Bach Mai Hospital Medical Collaboration Center, Hanoi, Viet Nam; 4 Department of Respiratory Medicine, National Center for Global Health and Medicine, Tokyo, Japan; 5 Hanoi Lung Hospital, Hanoi, Viet Nam; Fundació Institut d'Investigació en Ciències de la Salut Germans Trias i Pujol. Universitat Autònoma de Barcelona. CIBERES, Spain

## Abstract

**Background:**

Wasting is known as a prominent feature of tuberculosis (TB). To monitor the disease state, markers of metabolism and inflammation are potentially useful. We thus analyzed two major adipokines, adiponectin and leptin, and two other metabolic markers, fetuin-A and retinol-binding protein 4 (RBP4).

**Methods:**

The plasma levels of these markers were measured using enzyme-linked immunosorbent assays in 84 apparently healthy individuals ( = no-symptom group) and 46 patients with active pulmonary TB around the time of treatment, including at the midpoint evaluation ( = active-disease group) and compared them with body mass index (BMI), C-reactive protein (CRP), chest radiographs and TB-antigen specific response by interferon-γ release assay (IGRA).

**Results:**

In the no-symptom group, adiponectin and leptin showed negative and positive correlation with BMI respectively. In the active-disease group, at the time of diagnosis, leptin, fetuin-A and RBP4 levels were lower than in the no-symptom group [adjusted means 2.01 versus 4.50 ng/ml, *P*<0.0001; 185.58 versus 252.27 µg/ml, *P*<0.0001; 23.88 versus 43.79 µg/ml, *P*<0.0001, respectively]. High adiponectin and low leptin levels were associated with large infiltrates on chest radiographs even after adjustment for BMI and other covariates (*P* = 0.0033 and *P* = 0.0020). During treatment, adiponectin levels increased further and then decreased. Leptin levels remained low. Initial low levels of fetuin-A and RBP4 almost returned to the normal reference range in concert with reduced CRP.

**Conclusions:**

Our data and recent literature suggest that low fat store and underlying inflammation may regulate these metabolic markers in TB in a different way. Decreased leptin, increased adiponectin, or this ratio may be a promising marker for severity of the disease independent of BMI. We should further investigate pathological roles of the balance between these adipokines.

## Introduction

Tuberculosis (TB) is a major infectious cause of death around the world, with most of the 1.5 million deaths per year attributable to the disease occurring in developing countries. Negative energy balance in chronic inflammation has been recognized as a prominent feature of TB and one of the major obstacles to manage the patients [1], [2]. Recent emergence of drug resistant TB is assumed to be driven by poorly implemented drug regimens, but malnutrition as well as HIV co-infection might worsen the condition: Inflammatory responses evoked by infection increase the demand for anabolic energy, leading to a synergistic vicious circle and further deterioration of the clinical condition [3].

It is generally believed that undernourishment diminishes protective immunity against *Mycobacterium tuberculosis*. [4]. A series of animal experiments, particularly aerosol-infected guinea pig models have demonstrated that chronic protein-energy malnutrition reduces secretion of T-helper 1 (Th1) cytokines [5]. It is rapidly reversed with alimentary supplement, indicating a pivotal role of nutrition, although it remains unclear what the optimal nutritional interventions are for improving the human disease in an effective manner [4].

On the other hand, in many countries today, rapid industrialization and urbanization are accompanied by changing patterns of diet and physical activity and this results in overnutrition [6]. Consequently, a combination of these two unfavorable conditions, a slow decline of infectious diseases associated with undernutrition and a rapid increase in obesity and diabetes are a serious double burden to public health and clinical medicine in resource limited settings [7].

**Table 1:** Characteristics of study population.

	no-symptom group (N = 84)	active-disease group (N = 46)	*P* values
Male/Female (n)	41/43	42/4	<0.0001
Age (year)*	40.0 (28.1–48.6)	47.2 (34.7–55.0)	0.0064
BMI (kg/m^2^)*	21.8 (20.0–23.7)	18.3 (17.1–19.5)	<0.0001
BCG history (yes/no/unknown)	33/28/23	10/3/33	<0.0001
positive/negative results of IGRA (n)	55/29	41/4**	0.0015

* Median and 25-to-75 percentiles in parenthesis are shown.

** One indeterminate case is not shown here.

Mainly in studies carried out in industrialized countries, fat-cell-derived hormones/cytokines designated as adipokines and relevant mediators have been investigated extensively and proposed as markers of obesity and diabetes [8]. Of these adipokines, adiponectin is a unique insulin sensitizer with atheroprotective role [9], [10]. Plasma levels of adiponectin are inversely correlated with body weight and visceral fat mass [11], [12]. Leptin is another major adipokine in proportion to fat stores [13], [14] and one of the key mediators of energy metabolism [2] Even mild weight loss induced by dietary restriction is known to reduce leptin levels [11]. These markers supposedly shift towards the opposite in lean patients with wasting diseases. However, the significance of these metabolic markers in chronic infectious diseases like TB has not been fully understood [2].

**Table 2:** Correlation of tested marker levels with BMI, CRP and IGRA values in each of the no-symptom and active-disease groups.

	no-symptom group (N = 84)			active-disease group (N = 46)		
	Pearson's *r* (*P* values)^a^			Pearson's *r* (*P* values)^a^		
Variable	by BMI (kg/m^2^)	by CRP (µg/ml)	by IFN-γ (IU/ml)^b^	by BMI (kg/m^2^)	by CRP (µg/ml)	by IFN-γ (IU/ml)^b^
Adiponectin (µg/ml)	−0.4530	−0.2892	−0.2254	−0.4421	0.1477	−0.1092
	(<0.0001)*	(0.0076)	(0.0393)	(0.0021)	(0.3274)	(0.4700)
Leptin (ng/ml)	0.4518	0.1694	0.1179	0.2771	−0.0918	0.3568
	(<0.0001)*	(0.1234)	(0.2855)	(0.0623)	(0.5442)	(0.0149)
Leptin/adiponectin ratio	0.5820	0.2793	0.2067	0.4901	−0.1633	0.2804
	(<0.0001)*	(0.0101)	(0.0592)	(0.0005)*	(0.2783)	(0.0591)
Fetuin-A (µg/ml)	0.0309	0.0415	0.0322	0.1243	−0.1833	0.2402
	(0.7805)	(0.7079)	(0.7714)	(0.4105)	(0.2226)	(0.1078)
RBP4 (µg/ml)	0.1605	−0.0213	0.0716	0.1535	−0.3018	−0.0916
	(0.1447)	(0.8475)	(0.5173)	(0.3085)	(0.0415)	(0.5448)

a Pearson's correlation coefficients with *P* values were calculated. Plasma concentrations were analyzed after logarithmic transformation.

b TB-antigen stimulated IFN-γ response

* Statistically significant when the significance level is set as *P*<0.002 based on the Bonferrroni correction.

We have recently conducted a proteomic research and demonstrated that plasma levels of fetuin-A and retinol-binding protein 4 (RBP4), also closely linked to the metabolic and inflammatory state, were significantly lower in patients with active pulmonary TB than in control subjects [15]. Fetuin-A, also known as α2-Heremans-Schmid glycoprotein, is an abundant plasma component of hepatic origin [16] and a negative regulator of insulin signaling [17], [18]. Elevation of plasma fetuin-A is strongly associated with atherogenic lipid profile as well as fatty liver in obese patients [18]. Lipid components in the liver presumably upregulate fetuin-A expression, which may in turn repress adiponectin and impair adipocyte function [19], [20]. Fetuin-A is also downregulated in acute inflammation as a negative acute-phase protein [21]. RBP4, synthesized in the liver and adipose tissue, has recently been identified as another adipokine involved in the development of insulin resistance [22]. In humans, similar to leptin, circulating RBP4 levels are high in obesity and decreased after calorie-restriction induced weight loss [11], [23]. RBP4 is also known as a specific transporter protein for retinol (vitamin A) and can be used to assess the short-term fluctuation of nutritional states as a rapid turnover protein [24].

**Table 3:** BMI, CRP and tested marker levels in IGRA-positive and -negative subgroups in the no-symptom group.

	IGRA-negative (N = 29)		IGRA-positive (N = 55)		
marker	adjusted mean^a^	(95%CI)	adjusted mean^a^	(95%CI)	*P* values (ANCOVA)
BMI (kg/m^2^)	21.52	(20.58–22.46)	21.48	(20.74–22.22)	0.9392
CRP (µg/ml)	1.12	(0.60–2.08)	1.30	(0.80–2.12)	0.6663
Adiponectin (µg/ml)	7.19	(5.67–9.11)	6.39	(5.30–7.70 )	0.3792
Leptin (ng/ml)	4.50	(3.34–6.05)	4.38	(3.47–5.54)	0.8783
Leptin/adiponectin ratio	0.63	(0.40–0.97)	0.69	(0.49–0.97)	0.7080
Fetuin-A (µg/ml)	234.22	(212.40–258.29)	263.88	(244.26–285.06)	0.0333
RBP4 (µg/ml)	39.64	(32.28–48.69)	42.88	(36.45–50.43)	0.4997

a Estimated means of plasma concentrations were compared after logarithmic transformation, being adjusted for gender and age as covariates. The data shown are transformed back to the original unit.

No *P* values were statistically significant when the significance level is set as *P*<0.007 based on the Bonferrroni correction.

Alteration of the circulating levels of these markers should be investigated in TB, since they are expected to provide a basis of a critical link among nutritional status, metabolism and immunity of the disease, and hopefully to consider efficient nutritional interventions. In the present study, we thus measured circulating adiponectin and leptin in addition to fetuin-A and RBP4 levels in patients with active pulmonary TB versus apparently healthy individuals and compared the levels with body mass index (BMI), a simple estimate of adiposity [25] and C-reactive protein (CRP), a representative positive acute phase protein [26]. We further characterized their relationship with disease severity and alterations during the course of treatment.

**Table 4:** BMI, CRP and tested marker levels in the no-symptom and active-disease groups after adjustment for gender and age.

	no-symptom group (N = 84)		active-disease group (N = 46)		
marker	adjusted mean^a^	(95%CI)	adjusted mean^a^	(95%CI)	*P* values (ANCOVA)
BMI (kg/m^2^)	21.68	(21.06–22.30)	17.65	(16.66–18.65)	<0.0001*
CRP (µg/ml)	1.22	(0.86–1.74)	36.88	(20.94–64.94)	<0.0001*
Adiponectin (µg/ml)	6.82	(5.73–8.12)	9.29	(7.02–12.30)	0.0136
Leptin (ng/ml)	4.50	(3.78–5.35)	2.01	(1.52–2.66)	<0.0001*
Leptin/adiponectin ratio	0.66	(0.50–0.88)	0.22	(0.14–0.34)	<0.0001*
Fetuin-A (µg/ml)	252.27	(234.55–271.33)	185.58	(165.07–208.64)	<0.0001*
RBP4 (µg/ml)	43.79	(38.09–50.34)	23.88	(19.08–29.88)	<0.0001*

a Estimated means of plasma concentrations were compared after logarithmic transformation, being adjusted for gender and age as covariates. The data shown are transformed back to the original unit.

* Statistically significant when the significance level is set as *P*<0.007 based on the Bonferrroni correction.

## Methods

### Study design

We randomly selected and used plasma samples and demographic information in 46 patients with active pulmonary TB ( = active-disease group) without treatment history as a biomarker sub-study of a large cohort study [27]. All patients entered the study from July 2007 to March 2009. Diagnosis of active pulmonary TB was made clinically and radiologically and confirmed bacteriologically in Hanoi Lung Hospital. A sputum smear test showed positive results in all of the patients in the active disease group and all of them completed anti-TB treatment following the national standard regimen, 2 months of streptomycin, isoniazid, rifampicin, and pyrazinamide followed by 6 months of isoniazid and ethambutol (2SHRZ/6HE).

**Table 5:** CRP and tested marker levels in the no-symptom and active-disease groups after adjustment for gender, age and BMI.

	no-symptom group (N = 84)		active-disease group (N = 46)		
marker	adjusted mean^a^	(95%CI)	adjusted mean^a^	(95%CI)	*P* values (ANCOVA)
CRP (µg/ml)	1.11	(0.77–1.60)	47.80	(25.36–90.09)	<0.0001*
Adiponectin (µg/ml)	7.80	(6.63–9.19)	6.39	(4.81–8.49)	0.1671
Leptin (ng/ml)	3.77	(3.26–4.37)	3.28	(2.54–4.24)	0.2790
Leptin/adiponectin ratio	0.48	(0.38–0.61)	0.51	(0.35–0.76)	0.7704
Fetuin-A (µg/ml)	248.04	(229.95–267.57)	194.46	(170.48–221.80)	0.0004*
RBP4 (µg/ml)	42.90	(37.08–49.63)	25.27	(19.62–32.55)	0.0001*

a Estimated means of plasma concentrations were compared after logarithmic transformation, being adjusted for gender, age and BMI as covariates. The data shown are transformed back to the original unit.

* Statistically significant when the significance level is set as *P*<0.008 based on the Bonferrroni correction.

Chest radiographs were taken at the time of diagnosis and interpreted by two readers independently in a blind manner. The presence of cavitary lesions and the number of lung zones (zero to six corresponding to the upper, middle, and lower fields on the right and left sides of the lung) affected by infiltrates were recorded [28]. HIV status was examined before starting anti-TB treatment. The proportion of HIV co-infection is less than 10% in this study area and those with HIV positive were excluded from the drawing up of this sub-study.

**Table 6:** BMI, CRP and tested marker levels in patients with small and large infiltrates on chest radiographs after adjustment for gender and age.

	small infiltrates^a^ (N = 22)		large infiltrates^a^ (N = 23)		
marker	adjusted mean^b^	(95%CI)	adjusted mean^b^	(95%CI)	*P* values (ANCOVA)
BMI (kg/m^2^)	18.73	(16.74–20.71)	18.11	(15.95–20.27)	0.3065
CRP (µg/ml)	26.14	(12.63–54.10)	35.92	(16.29–79.21)	0.1520
Adiponectin (µg/ml)	10.28	(5.38–19.66)	18.83	(9.31–38.11)	0.0033*
Leptin (ng/ml)	2.42	(1.64–3.57)	1.65	(1.08–2.52)	0.0020*
Leptin/adiponectin ratio	0.24	(0.11–0.52)	0.09	(0.04–0.21)	0.0002*
Fetuin-A (µg/ml)	201.97	(149.87–272.18)	184.68	(133.52–255.46)	0.3222
RBP4 (µg/ml)	36.14	(21.76–60.03)	31.56	(18.17–54.79)	0.3770
IFN-γ (IU/ml)^c^	11.04	(2.13–57.16)	5.80	(0.97–34.82)	0.2039

a Small infiltrates = less than 3 of 6 zones in the lung affected, large infiltrates = 3 or more than 3 of 6 zones affected

b Estimated means of plasma concentrations were compared after logarithmic transformation, being adjusted for gender and age as covariates. The data shown are transformed back to the original unit.

c TB-antigen stimulated IFN-γ response

* Statistically significant when the significance level is set as *P*<0.006 based on the Bonferrroni correction.

As a reference, we also measured plasma samples derived from 84 apparently healthy men and women who may have chances of direct or indirect contacts with TB patients as health care staff ( = no-symptom group). All participants were tested for TB-antigen specific interferon-γ response by the commercially available enzyme-linked immunosorbent assay (ELISA)-based interferon-γ release assay (IGRA), QuantiFERON-TB Gold In-Tube™ (Cellestis, Victoria, Australia). In the no-symptom group, IGRA-positive individuals suspected of latent TB infection were recommended to take chest radiography and to confirm there were no active pulmonary lesions. Subsequently a chance of receiving isoniazid prophylactic therapy was given. The protocol was approved by ethical committees of the Ministry of Health, Viet Nam and National Center for Global Health and Medicine, Japan respectively and written informed consent was obtained from each participant.

**Table 7:** CRP and tested marker levels in patients with small and large infiltrates on chest radiographs after adjustment for gender, age and BMI.

	small infiltrates^a^ (N = 22)		large infiltrates^a^ (N = 23)		
marker	adjusted mean^b^	(95%CI)	adjusted mean^b^	(95%CI)	*P* values (ANCOVA)
CRP (µg/ml)	26.59	(12.78–55.28)	35.50	(16.02–78.63)	0.1991
Adiponectin (µg/ml)	10.84	(6.01–19.53)	18.15	(9.57–34.40)	0.0061*
Leptin (ng/ml)	2.37	(1.63–3.47)	1.67	(1.11–2.52)	0.0040*
Leptin/adiponectin ratio	0.22	(0.11–0.44)	0.09	(0.04–0.20)	0.0002*
Fetuin-A (µg/ml)	200.77	(148.59–271.28)	185.46	(133.74–257.18)	0.3886
RBP4 (µg/ml)	35.69	(21.43–59.46)	31.83	(18.29–55.42)	0.4626
IFN-γ (IU/ml)^c^	11.41	(2.17–59.90)	5.68	(0.94–34.53)	0.1760

a Small infiltrates = less than 3 of 6 zones in the lung affected, large infiltrates = 3 or more than 3 of 6 zones affected

b Estimated means of plasma concentrations were compared after logarithmic transformation, being adjusted for gender, age and BMI as covariates. The data shown are transformed back to the original unit.

c TB-antigen stimulated IFN-γ response

* Statistically significant when the significance level is set as *P*<0.007 based on the Bonferrroni correction.

### Measurements of markers of metabolism and inflammation

Immediately after making the diagnosis of active TB disease, heparinized blood samples were drawn for IGRA before starting anti-TB treatment (0 month) and the remaining plasma without mixing any stimulants was reserved in a −80°C freezer until measurement. Samples were collected twice again, after the initial phase of treatment (2 months) and at the end of treatment (7 months) in the active disease group. This study was originally intended to identify a variety of biomarkers associated with TB phenotypes [15] and the participants were not obliged to keep fasting. The blood was collected in the daytime between 8 am and 4 pm at the outpatient clinic to avoid interference in dosing schedule of anti-TB drugs.

**Figure 1:**
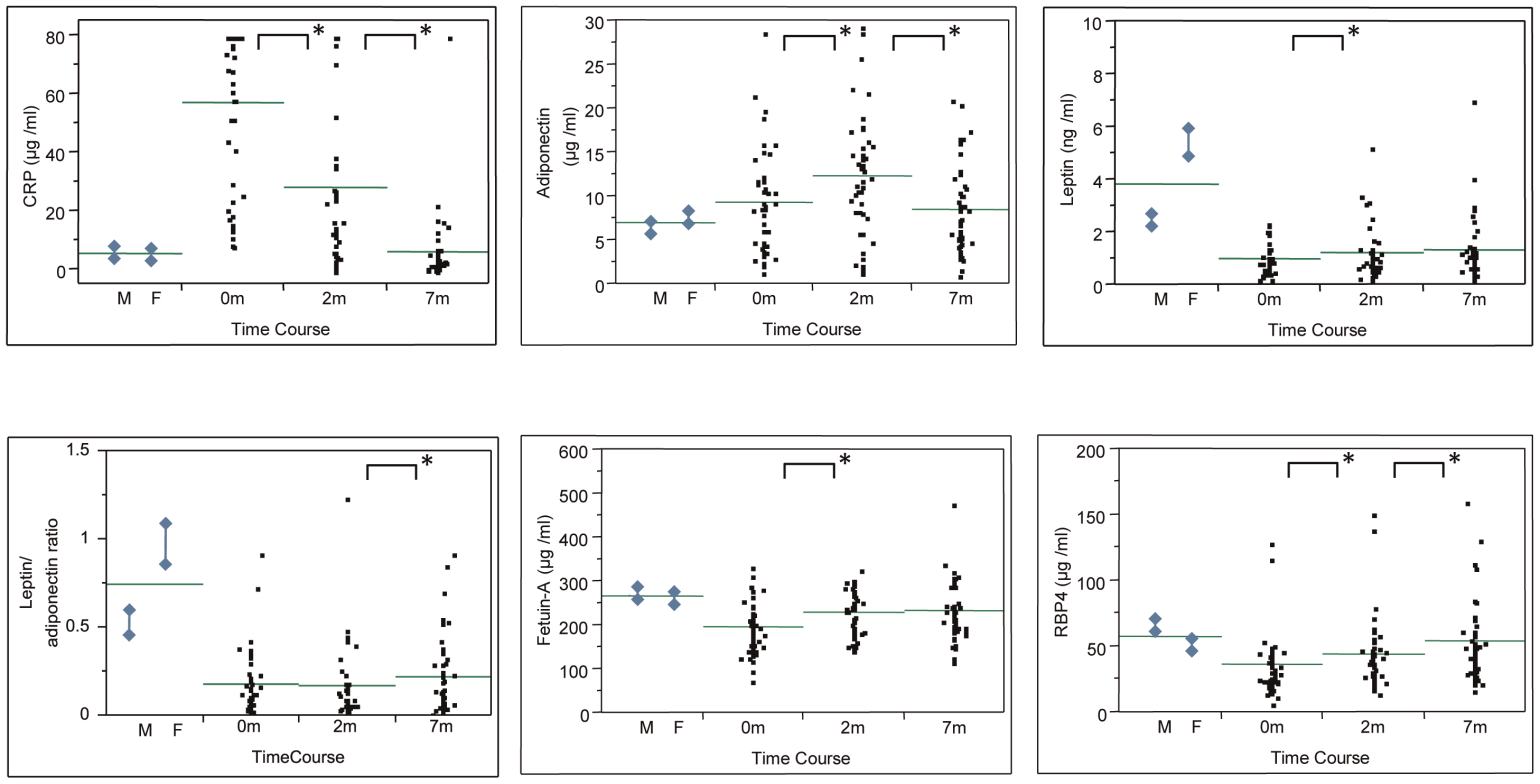
CRP and tested marker levels in patients with active TB before (0 month), during (2 months) and at the end (7 months) of anti-TB treatment (N = 46). Vertical bars with diamonds on the left side (M and F) indicate reference values, means ± SEM of the values in men (N = 41) and women (N = 43) of the no-symptom group. A horizontal bar indicates the grand mean of the values in each condition. * indicates *P*<0.05 by paired comparison between 0 month and 2 months. When significant, 2 months and 7 months were also compared.

The AssayMax Human C-Reactive Protein ELISA kit was used for detection of human c-reactive protein (CRP) in plasma (Assaypro LLC. St. Charles, MO, USA). The minimum detectable dose was less than 0.25 ng/ml. The Quantikine® Human Total Adiponectin/Acrp30 Immunoassay kit was used to detect total (low, middle and high molecular weight) human adiponectin in plasma (R&D Systems, Inc.; Minneapolis, MN, USA). The mean minimum detectable dose was 0.246 ng/ml. The Quantikine® Human Leptin Immunoassay kit was used to detect human leptin in plasma (R&D Systems, Inc.). The mean minimum detectable dose was 7.8 pg/ml. The AHSG ELISA kit was used to detect fetuin-A in plasma (BioVender Laboratory Medicine Inc.; Modrice, Czech Republic). The detection limit was 0.35 ng/ml. A competitive ELISA for quantitative determination of RBP4 in human plasma was also applied (AdipoGen Inc.; Seoul, Korea) and the detection limit was 1 ng/ml. All were performed according to the manufacturer's instructions. Differences in measured concentrations between EDTA plasma samples as reference and these heparin samples were within a range of variation generally accepted in ELISA (coefficient of variance <15%) (data not shown)

### Statistical analysis

Plasma protein levels were served for subsequent statistical analysis after logarithmic transformation of the measurements to minimize distortion of the data distribution. Means of demographic data between two groups were compared by analysis of variance (ANOVA) after testing for equal variances and proportions between two groups were compared by the chi-squared test. Since it is well known that levels of adipokines such as leptin are influenced by gender and age, measurements of protein markers in any two groups were compared by analysis of covariance (ANCOVA) to allow for the covariates. The relationship between markers and other parameters were assessed by Pearson's correlation coefficients. Overall alterations of the measurements at three time points were initially analyzed by repeated-measures ANOVA and only when statistically significant, post-hoc comparisons were proceeded to: Difference of values between two time points was assessed by the paired-T test, under normal approximation based on the central limit theorem. *P* values<0.05 were considered to be statistically significant in general. When the Bonferroni correction was applied, however, a level of statistical significance was set as 0.05/n (n = the number of comparisons). Statistical analysis was performed using Stata version 11 (StataCorp, College Station, TX, USA).

## Results

### Characteristics of study population

The no-symptom group consisted of 84 apparently healthy individuals, whose blood samples were used to obtain the standard values of markers in the study population. This group includes an approximately equal number of men and women with median age of 40, and more than half of the individuals had latent TB infection diagnosed by the IGRA method (Table 1). The active-disease group members were 46 patients with smear-positive active pulmonary TB. The majority of the patients were male with low body mass index (BMI<18.5 kg/m^2^) and the median age was 47, slightly older than in the non-symptom group.

### Correlation of adiponectin, leptin, fetuin-A and RBP4 levels with BMI, CRP and IGRA values in the no-symptom and active-disease groups

Correlation coefficients (*r*) were calculated in the no-symptom and active-disease groups respectively (Table 2). Adiponectin and leptin showed negative and positive correlations with BMI respectively in the no-symptom group (*r* = −0.4530, *P*<0.0001; *r* = 0.4518, *P*<0.0001). Leptin/adiponectin ratio showed a positive correlation with BMI in the active-disease group (*r* = 0.4901, *P* = 0.0005) as well as in the no-symptom group (*r* = 0.5820, *P*<0.0001). These correlations were statistically significant even after Bonferroni correction for multiple comparisons. The other possible correlations including a pair of leptin and TB-antigen stimulated IFN-γ response did not reach significant levels in this study, when Bonferroni correction was applied.

### Pairwise correlations between four tested markers

Pairwise correlation coefficients (*r*) between four tested metabolic markers were further calculated in the no-symptom and active-disease groups respectively (Table S1). A significant correlation was found only between fetuin-A and RBP4 levels (*r* = 0.4007, *P* = 0.0058) in the active disease group.

### Adiponectin, leptin, fetuin-A and RBP4 levels with IGRA-positive and -negative subgroups in the no-symptom group

IGRA-positive values higher than the cutoff value, 0.35 IU/ml are regarded as latent TB infection after active disease is ruled out. We thus categorized the no-symptom group into IGRA-positive and -negative subgroups and compared plasma concentrations of the above markers. However, none of the marker levels including fetuin-A were significantly different between IGRA-positive and -negative subgroups after adjustment for gender and age, when considering the number of comparisons (Table 3).

### Adiponectin, leptin, fetuin-A and RBP4 levels in the no-symptom and active-disease groups

The active-disease group had significantly low BMI and very high CRP levels at the time of diagnosis, when assessed by using ANCOVA with adjusted means (Table 4). In the disease group, leptin, leptin/adiponectin ratio, fetuin-A and RBP4 levels were remarkably lower than in the no-symptom group (*P*<0.0001 respectively) after adjustment for gender and age and these differences were statistically significant even after Bonferroni correction (Table 4).

Since BMI was strongly correlated with some of the adipokine values as shown in Table 2, we further analyzed levels of the four markers after adjustment for BMI as well as gender and age. Consequently, adiponectin and leptin levels were not significantly different between the two groups any more, whereas fetuin-A and RBP4 levels remained significant (*P* = 0.0004 and *P* = 0.0001) (Table 5)

### Adiponectin, leptin, fetuin-A and RBP4 levels in patients with mild and severe disease

At the time of diagnosis, severity of the disease was assessed by spread of infiltrates on chest radiographs (Table 6). Small infiltrates affecting less than 3 of the 6 lung zones and large ones affecting more, categorized the patients into two subgroups ( = mild and severe disease) half-and-half.

After adjustment for gender and age, adiponectin levels were higher and leptin levels were lower in patients with large infiltrates than in those with small infiltrates (*P* = 0.0033 and *P* = 0.0020). Interestingly, differences in the levels of these two adipokines between small and large infiltrates were significant respectively (*P* = 0.0061 and *P* = 0.0040), even after adjustment for BMI as well as gender and age (Table 7). Leptin/adiponectin ratio was lower, or adiponectin/leptin ratio was higher, in patients with large infiltrates than in those with small infiltrates independent of BMI (*P* = 0.0002). None of the markers were associated with the presence of cavity on the chest radiographs (data not shown).

### Adiponectin, leptin, fetuin-A and RBP4 levels in patients with active TB before, during and at the end of anti-TB treatment

Figure 1 shows plasma values at the time points before (0 month), during (2 months) and at the end (7 months) of anti-TB treatment. Mean values in men (N = 41) and women (N = 43) of the no-symptom group are shown as a reference, in which gender difference was observed in leptin levels and leptin/adiponectin ratio (*P*<0.0001).

Overall differences of the measurements during anti-TB treatment in all of these four markers were statistically significant by repeated-measures ANOVA (*P*<0.01). Post-hoc analysis showed that adiponectin levels increased transiently (*P* = 0.0004; 0 month vs. 2 months) and then decreased close to the reference range by the end of treatment (*P*<0.0001; 2 months vs. 7 months). Leptin levels remained low throughout the treatment course, though gradually elevated (*P* = 0.0226; 0 month vs. 2 months). Initial low levels of fetuin-A and RBP4 significantly improved during treatment (*P* = 0.0001 and *P* = 0.0016; 0 month vs. 2 months), almost reaching the reference range by the end in concert with reduced CRP levels.

## Discussion

We assessed the clinical significance of four metabolic markers, adiponectin, leptin, fetuin-A and RBP4 in patients with active TB, analyzing them in relation to classical nutritional and inflammatory parameters, BMI and CRP, severity of disease and treatment course. BMI is known to be lower in patients with active TB than in control subjects [1], [2]. After effective treatment, weight often increases but patients may remain underweight [11].

Plasma levels of adiponectin were inversely correlated with BMI in concordance with previous results [11], [12]. The adiponectin levels tended to be elevated in the active-disease group characterized by low BMI, though it did not reach significant levels, which was also shown by others [29]. Interestingly in our study, adiponectin levels were significantly higher in severe disease with extensive pulmonary lesions than in mild disease, even after adjustment for BMI. Adiponectin as a modulator of inflammation in a variety of diseases has recently been highlighted [30]. For instance, in critically ill patients, adiponectin levels appear to be transiently suppressed at the initial phase and then gradually elevated at the recovery phase [31], [32]. The plasma concentrations in patients with active TB were further increased after starting treatment and then decreased close to the reference range by the end of treatment. Elevated adiponectin levels in chronic inflammatory diseases may be explained by compensatory response to the underlying disease as well as concomitant low body fat mass, which is postulated by others [33], [34]. A study designed to measure alteration of adiponectin and BMI simultaneously throughout the treatment period would be able to characterize it further.

In most recent reports, leptin levels are low in TB [29], [35]–[38], though other earlier or smaller studies have shown conflicting results [39]–[42]. In the present study, using a commercial ELISA, significantly lower levels of leptin were demonstrated in patients with active TB, which could be mostly explained by marked undernutrition in our disease population. Within the active-disease group, however, correlation between leptin and BMI was less clear. BMI-independent regulation of plasma leptin concentrations should also be taken into consideration in TB at least in part [13], [37]. This idea is also supported by an *ex vivo* study by others demonstrating that continuous exposure of IL-1 or TNF-α provides a signal to downregulate leptin in human adipose tissue [43], though acute inflammation such as sepsis may rather upregulate circulating leptin levels transiently [44]–[46]. In addition to relatively high levels of adiponectin, low levels of leptin were observed in patients with large infiltrates, even after adjustment for BMI. This is concordant with a recent study showing that leptin levels were low in severe TB disease [29]. We have further demonstrated that low leptin/adiponectin ratio, or high adiponectin/leptin ratio is characteristic to severe TB disease in this study. This ratio was originally proposed as an atherogenic index indicating a balance between the two markers bearing apparently opposite functions in inflammation [47]. Our findings support the idea that suppressed production of leptin may be detrimental to host defense against TB by virtue of impairment of Th1 cell-mediated immunity [13], [29], [48]. After starting treatment, leptin levels were slightly elevated, but remained low during the treatment period. This is also compatible with reports made by others [37], [38], although the mechanism remains unknown. Long-lasting low levels of leptin may be attributed to individual predisposition to TB or delayed recovery from wasting disease.

In our study, fetuin-A levels were considerably low in TB even after adjustment for BMI. Soon after starting treatment, the levels were increased in inverse proportion to the decrease in CRP. In TB, fetuin-A may be downregulated by at least dual mechanisms, strongly mediated by underlying inflammation [21] and partly controlled by depleted liver fat due to wasting or malnutrition [18]. Low fetuin-A levels may also result in impairment of macrophage function to kill the pathogen and ectopic calcification possibly in TB lesions [49], [50].

RBP4 levels were also low in TB even after adjustment for BMI. Throughout the treatment course, the levels were gradually elevated close to the reference range inversely with the decrease in CRP. These findings are supported by a recent report demonstrating that RBP4 rapidly decreases during acute inflammation, possibly acting as a negative acute phase reactant, similar to fetuin-A, albumin and prealbumin [21], [51], [52]. This may partly explain a close positive correlation with fetuin-A demonstrated in the active-disease group. In addition to dual regulation of RBP4 by underlying inflammation and low body fat mass, reduced renal function is also known to cause retention of the circulating levels, such that further caution is needed to interpret RBP4 measurement in disease state [53].

Our study has several limitations. Firstly, many types of nutrients including micronutrients are essential to the human body but the potential interplay between each component of nutrients was not within our scope at that time. Secondly, since change of BMI was not measured during treatment, direct comparison of improved BMI with the corresponding marker levels was not possible. Thirdly, blood was collected during the daytime without enforced fasting. Although, of course, this increases the variance of measurements, it can be inferred that daytime variations on circulating adipokines and leptin [54] are not as large as to seriously affect conclusive results of comparisons within and between groups in this study. Finally, computer tomography, which has advantages over chest radiography as an imaging tool, was not available in our setting.

Overall, our data and recent literature would suggest that all of the four markers tested are controlled partly by low fat store and partly by inflammation in TB but their regulatory mechanisms are more or less different and interactions with other relevant factors including insulin sensitivity and cellular immunity are worth further investigation. In particular, leptin, adiponectin and their ratio may be promising markers for severity of the wasting disease. Since nutritional intervention has a potential to improve prognosis of intractable TB such as HIV co-infection and MDR-TB, large-scale prospective studies using selected biomarkers to investigate metabolic contributors to disease phenotype are desired. The more fully we understand the mechanisms linking diet, health, and disease, the more effective will be our ability to design optimal interventions.

## Supporting Information

Table S1 Pairwise correlations between four tested markers.
(DOC)
