# Supplementary material for: Circulating Levels of Adiponectin, Leptin, Fetuin-A and Retinol-Binding Protein in Patients with Tuberculosis: Markers of Metabolism and Inflammation
Source: PLoS One. 2012 Jun 7;7(6):e38703. doi: 10.1371/journal.pone.0038703 (PMC3369865; doi:10.1371/journal.pone.0038703)
Supplement: Table S1 — Pairwise correlations between four tested markers. (DOC) [file pone.0038703.s001.doc]

| **Table S1:** Pairwise correlations between four tested markers^a^ | | | | | |
| --- | --- | --- | --- | --- | --- |
| no-symptom group (N=84) |  | Adiponectin (μg/ml) | Leptin (ng/ml) | Fetuin-A (μg/ml) | rRBP4 (μg/ml) |
| Adiponectin (μg/ml) |  | 1.0000 |  |  |  |
|  |  |  |  |  |  |
|  |  |  |  |  |  |
| Leptin (ng/ml) |  | -0.1729 | 1.0000 |  |  |
|  |  | (0.1159) |  |  |  |
|  |  |  |  |  |  |
| Fetuin-A (μg/ml) |  | -0.1049 | -0.0754 | 1.0000 |  |
|  |  | (0.3421) | (0.4952) |  |  |
|  |  |  |  |  |  |
| RBP4 (μg/ml) |  | -0.2580 | -0.1506 | 0.1205 | 1.0000 |
|  |  | (0.0178) | (0.1716) | (0.2747) |  |
|  |  |  |  |  |  |
|  |  |  |  |  |  |
| active-disease group (N=46) |  | Adiponectin (μg/ml) | Leptin (ng/ml) | Fetuin-A (μg/ml) | rRBP4 (μg/ml) |
| Adiponectin (μg/ml) |  | 1.0000 |  |  |  |
|  |  |  |  |  |  |
|  |  |  |  |  |  |
| Leptin (ng/ml) |  | -0.1349 | 1.0000 |  |  |
|  |  | (0.3713) |  |  |  |
|  |  |  |  |  |  |
| Fetuin-A (μg/ml) |  | -0.2932 | 0.2220 | 1.0000 |  |
|  |  | (0.0479) | (0.1382) |  |  |
|  |  |  |  |  |  |
| RBP4 (μg/ml) |  | -0.2397 | 0.1126 | 0.4007 | 1.0000 |
|  |  | (0.1086) | (0.4561) | (0.0058)* |  |
|  |  |  |  |  |  |

^a^Pearson’s correlation coefficients with *P* values were calculated. Plasma concentrations were analyzed after logarithmic transformation.

*Statistically significant when the significance level is set as *P*< 0.008 based on the Bonferrroni correction.
